# Re-evaluation of glycerol utilization in *Saccharomyces cerevisiae*: characterization of an isolate that grows on glycerol without supporting supplements

**DOI:** 10.1186/1754-6834-6-157

**Published:** 2013-11-08

**Authors:** Steve Swinnen, Mathias Klein, Martina Carrillo, Joseph McInnes, Huyen Thi Thanh Nguyen, Elke Nevoigt

**Affiliations:** 1 School of Engineering and Science, Jacobs University Bremen gGmbH, Campus Ring 1, 28759 Bremen, Germany

**Keywords:** Yeast, *Saccharomyces cerevisiae*, Glycerol, *STL1*, *GUT1*, *GUT2*

## Abstract

**Background:**

Glycerol has attracted attention as a carbon source for microbial production processes due to the large amounts of crude glycerol waste resulting from biodiesel production. The current knowledge about the genetics and physiology of glycerol uptake and catabolism in the versatile industrial biotechnology production host *Saccharomyces cerevisiae* has been mainly based on auxotrophic laboratory strains, and carried out in the presence of growth-supporting supplements such as amino acids and nucleic bases. The latter may have resulted in ambiguous conclusions concerning glycerol growth in this species. The purpose of this study was to re-evaluate growth of *S. cerevisiae* in synthetic glycerol medium without the addition of supplements.

**Results:**

Initial experiments showed that prototrophic versions of the laboratory strains CEN.PK, W303, and S288c did not exhibit any growth in synthetic glycerol medium without supporting supplements. However, a screening of 52 *S. cerevisiae* isolates for growth in the same medium revealed a high intraspecies diversity. Within this group significant variation with respect to the lag phase and maximum specific growth rate was observed. A haploid segregant of one good glycerol grower (CBS 6412-13A) was selected for detailed analysis. Single deletions of the genes encoding for the glycerol/H^+^ symporter (*STL1*), the glycerol kinase (*GUT1*), and the mitochondrial FAD^+^-dependent glycerol 3-phosphate dehydrogenase (*GUT2*) abolished glycerol growth in this strain, implying that it uses the same glycerol utilization pathway as previously identified in auxotrophic laboratory strains. Segregant analysis of a cross between CBS 6412-13A and CEN.PK113-1A revealed that the glycerol growth phenotype is a quantitative trait. Genetic linkage and reciprocal hemizygosity analysis demonstrated that *GUT1*_*CBS 6412-13A*_ is one of the multiple genetic loci contributing to the glycerol growth phenotype.

**Conclusion:**

The *S. cerevisiae* intraspecies diversity with regard to glycerol growth is a valuable starting point to identify the genetic and molecular basis of this phenotype. This knowledge can be applied for further rational strain improvement with the goal of using glycerol as a carbon source in industrial biotechnology processes based on *S. cerevisiae* as a production organism.

## Background

Biodiesel and bioethanol have been preferred solutions in order to meet Europe’s ambitious goals for reducing greenhouse gas emissions. Particularly during biodiesel production glycerol is an inevitable by-product. In fact, biodiesel production, that is, the transesterification of fats and oils with an alcohol, results in 10 lbs of crude glycerol for every 100 lbs of biodiesel produced [1]. The vast growth of biofuel industries over the past few years has led to a dramatic surplus of crude glycerol accompanied by a decrease in its price [1,2]. As it is not cost-effective to purify glycerol from waste streams for use in food, pharmaceutical, or cosmetics industries, alternative avenues for crude glycerol valorization have been evaluated with a focus on microbial conversions [3,4]. Indeed, many microorganisms are able to utilize glycerol as the sole source of carbon [5]. Moreover, such microorganisms can, at least to a certain extent, tolerate the impurities present in crude glycerol [4,6]. Thus, the use of crude glycerol as feedstock for bioprocesses based on microbial fermentations has emerged as an attractive idea.

An integrated biorefinery concept alongside biodiesel production requires platform cell factories specifically suited to produce various valuable products from waste glycerol at industrial scale, such as commodity and high value fine chemicals as well as recombinant proteins. Several such production processes have already been established or envisaged [1,4,7,8].

The yeast species *Saccharomyces cerevisiae* is a popular platform in metabolic engineering as well as an attractive production organism in industrial biotechnology. This status is the result of factors such as the intense experience with this organism in industrial fermentations, the ease of genetic engineering, and its robustness under process conditions. There have been numerous metabolic engineering efforts aiming at the production of the whole range of industrially relevant products such as biofuels, bulk and fine chemicals (including pharmaceuticals), as well as protein drugs [9,10]. Some of these have already been commercialized while others are in the pipeline. However, *S. cerevisiae* only grows poorly on glycerol as a carbon source, and therefore it is evident that a substantial improvement of glycerol utilization in this species is of great commercial interest.

Barnett *et al*. [11] reported a high intraspecies diversity of *S. cerevisiae* with regard to glycerol growth but no quantitative data have been available so far. When carefully surveying the literature, it becomes evident that virtually all previous studies concerning glycerol growth of *S. cerevisiae* have been performed in the presence of supplements which deliberately or non-deliberately supported the growth. Examples for such supplements are 0.05% peptone [12,13], 0.1% yeast extract and 0.075% bacto peptone [14], 1% yeast extract and 2% peptone [15], or 0.2% glucose as a starter substrate [16,17]. In addition, all genetic and molecular biology studies regarding glycerol uptake and dissimilation in *S. cerevisiae* have been carried out in laboratory strains carrying multiple auxotrophies. Although few of these studies applied synthetic medium [18,19], the studied (auxotrophic) strains still required the addition of multiple medium supplements such as amino acids and nucleic bases. In fact, there have been indications that commonly used *S. cerevisiae* strains do not grow in synthetic glycerol medium, and that complex supplements are a requirement or at least support glycerol growth [20,21].

It is generally accepted that the major pathway of glycerol catabolism in *S. cerevisiae* is encoded by three genes: *STL1* (encoding a glycerol/H^+^ symporter), *GUT1* (encoding a glycerol kinase), and *GUT2* (encoding a FAD^+^-dependent glycerol 3-phosphate dehydrogenase localized to the outer leaflet of the inner mitochondrial membrane). The removal of any single one of these gene products by mutation or deletion in laboratory strains resulted in an almost complete abolishment of glycerol growth [12,19]. This result also implied that potential alternative glycerol catabolic pathways such as the dihydroxyacetone (DHA) pathway known from yeast species such as *Schizosaccharomyces pombe*[22] cannot have a significant importance for glycerol utilization in *S. cerevisiae,* although it has been recently shown that this pathway might be functional under certain conditions [23]. Similarly, alternative transporters such as Fps1 [24], Gup1, and Gup2 [25] do not seem to be significantly involved in glycerol uptake during growth of *S. cerevisiae* on glycerol. However, it should be emphasized that the conclusions about the major pathway of glycerol uptake and catabolism have been based on mutants of laboratory strains carrying multiple auxotrophic markers and thus requiring the addition of appropriate medium supplements [12,19,24,25].

This study aimed at evaluating the intraspecies diversity of *S. cerevisiae* with regard to growth on glycerol as the sole source of carbon as this is a key for the better understanding of the molecular basis underlying glycerol utilization in this organism. After identification of *S. cerevisiae* isolates able to grow on glycerol even without the addition of any supplements, we characterized one haploid segregant of a good glycerol grower (referred to here as the glycerol^+^ strain) in more detail. We demonstrate that the *GUT1* allele from the glycerol^+^ strain is one of the multiple genetic determinants for this phenotype.

## Results

### Common laboratory strains of *S. cerevisiae* cannot grow in synthetic glycerol medium without supplements

The diversity of media, supplements, and strains used to characterize glycerol utilization and growth of *S. cerevisiae* impedes a concluding evaluation of this trait by literature survey alone. Recent studies indicate that commonly used laboratory strains of *S. cerevisiae* do not grow in synthetic medium containing glycerol as the sole source of carbon [20,21]. In order to evaluate the impact of supplements on growth characteristics of laboratory strains in synthetic medium, three representative and well-characterized laboratory strains (CEN.PK113-7D, W303-1A, and S288c) were analyzed. For all strains prototrophic variants were used to avoid the necessity of supplementing the media with amino acids and/or nucleic bases.

**Figure 1:**
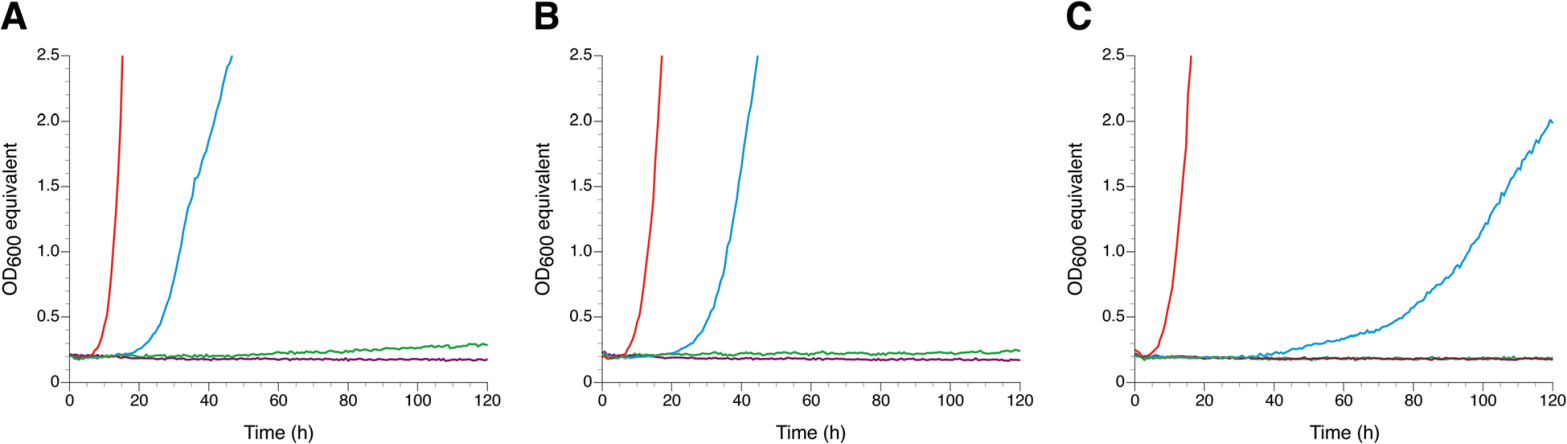
**Effect of supplements on the glycerol growth of *S. cerevisiae *laboratory strains.** Cells of **(A)** CEN.PK113-7D, **(B)** W303-1A, and **(C)** S288c were pre-cultivated in synthetic glucose medium for 48 hours. Afterwards, an appropriate number of cells were washed once in either synthetic glucose medium (red), synthetic glycerol medium (green), synthetic glycerol medium supplemented with CSM (blue), or synthetic medium without any carbon source but supplemented with CSM (purple), and then used for inoculating the respective medium at an optical density of 0.2. The cultures were cultivated in the Growth Profiler as described in the Methods section. Data from one representative experiment out of three independent biological replicates are shown.

The three tested *S. cerevisiae* laboratory strains did not grow in synthetic medium containing 6% (v/v) glycerol (Figure 1). When glycerol was replaced by 2% (w/v) glucose all strains grew after a short lag phase. Supplementation of synthetic glycerol medium with CSM (a ready-to-use mixture of amino acid and nucleic base supplements) resulted in growth of all three strains, albeit at a lower growth rate and after a longer lag phase as compared to synthetic glucose medium. The sole addition of CSM to the synthetic medium without any carbon source did not result in growth demonstrating that the amino acids and nucleic bases cannot serve as carbon sources *per se* but rather support the utilization of glycerol as a carbon source.

### Intraspecies diversity of *S. cerevisiae* strains with regard to glycerol growth

**Figure 2:**
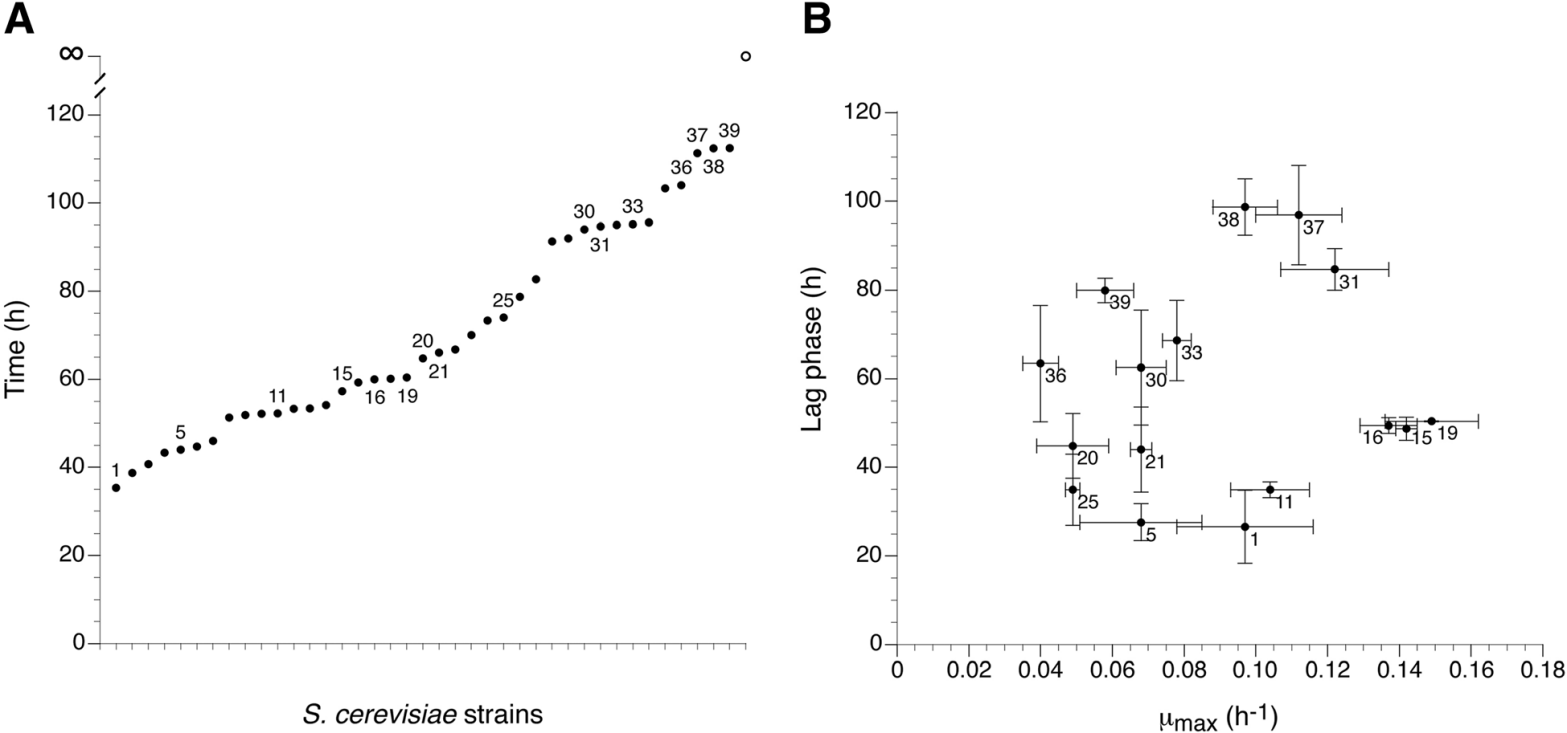
**Phenotypic intraspecies diversity of *S. cerevisiae *regarding growth on glycerol as the sole source of carbon.** A total of 52 strains of *S. cerevisiae* were screened for growth in synthetic glycerol medium (without the addition of amino acids or nucleic bases) using the Growth Profiler. A list of all the strains including the strain name and source is given in Additional file 1. **(A)** For all strains, the time needed to reach an OD_600_ of 1 is shown. Out of the 52 strains, 13 did not show growth during the course of the experiment (represented by the open symbol at time ∞). **(B)** Scatterplot of μ_max_ versus lag phase for 16 selected strains. Strain CBS 6412 has been assigned number 1 in both plots. Mean values and standard deviations were obtained from three biological replicates.

*S. cerevisiae* is known to exhibit a high phenotypic and genotypic intraspecies diversity [26-32], which implies that also varying glycerol growth phenotypes may exist in this species. In industrial bioprocesses using growing cells as cell factories, the velocity of biomass formation is an important parameter for volumetric productivity. Therefore, in a preliminary screening, we used the time required to reach an optical density (OD_600_) of 1 in synthetic glycerol medium to compare the biomass formation of 52 different *S. cerevisiae* strains (Figure 2A). Most of the strains originate from the Culture Collection of Extremophilic Fungi (EXF; Infrastructural Centre Mycosmo, Department of Biology, Biotechnical Faculty, University of Ljubljana, Slovenia), and a list of these strains is given in Additional file 1. Our data confirmed considerable intraspecies diversity in terms of glycerol growth. Out of the 52 strains, 13 did not grow throughout the course of the experiment (120 hours). This group of non-growers (hereafter referred to as the glycerol^-^ strains) includes a diploid CEN.PK laboratory strain and the well-known industrial strains Ethanol Red, Thermosacc Dry, and Alcotec 24. The strain which required the least time to reach an optical density of 1 was CBS 6412.

As biomass formation is affected by both growth rate and lag phase, 16 strains (including CBS 6412) covering the whole range of glycerol growth phenotypes were selected for detailed analysis of both parameters (Figure 2B). Within this group, no correlation between the maximum specific growth rate (μ_max_) and lag phase was observed. Although the μ_max_ of CBS 6412 was not among the highest values observed, we selected this strain for further characterization as it combines the shortest lag phase (27 hours) with a relatively high μ_max_ (0.10 h^-1^). The decision to focus on this strain was supported by the fact that it is known to be heterothallic, to show a good sporulation capacity, and to produce viable spores [33,34]. According to the CBS strain collection (CBS-KNAW Fungal Biodiversity Centre, The Netherlands), this strain has been isolated from sake brewing.

### Isolation of a haploid segregant from the glycerol^+^ strain CBS 6412 with comparable glycerol growth

As detailed molecular and genetic studies underlying the glycerol^+^ phenotype are strongly facilitated by analyzing a haploid strain, CBS 6412 was sporulated and haploid segregants exhibiting the same glycerol^+^ phenotype were isolated. In total, 91 segregants were prescreened on solid synthetic glycerol medium. Out of these 91, 50 segregants showed little or no detectable growth after incubation for 5 days. The remaining segregants either showed intermediate growth (35 segregants), or grew as well as CBS 6412 (6 segregants). The growth characteristics of the six superior segregants were then characterized in more detail in liquid cultures (data not shown). Segregant CBS 6412-13A was selected for further studies since it showed a lag phase and μ_max_ comparable to the diploid parent CBS 6412 (data not shown).

### Re-evaluation of the major glycerol catabolic pathway in a glycerol^+^ strain: characterization of *stl1*∆, *gut1*∆, and *gut2*∆ mutants derived from CBS 6412-13A

**Figure 3:**
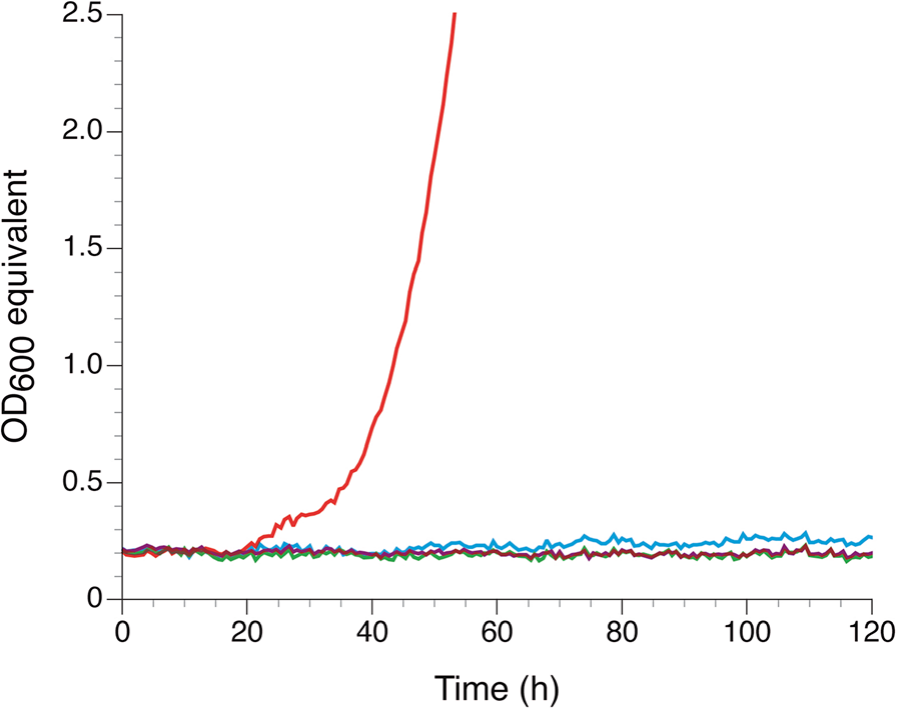
**Glycerol growth in synthetic medium of strains carrying deletions in the glycerol catabolic pathway derived from CBS 6412-13A.** Strains deleted for *GUT1* (green), *GUT2* (purple), and *STL1* (blue) did not show any growth. The haploid CBS 6412-13A (red) was included for comparison. Representative data from one of three independent experiments are shown.

Based on previous studies using laboratory strains it has been concluded that Stl1, Gut1, and Gut2 comprise the major glycerol catabolic pathway in *S. cerevisiae*. In contrast to the strains used in these studies, CBS 6412 grows in synthetic glycerol medium even without supplements. As it could not be excluded that this strain contains genes encoding for additional glycerol transporters and/or glycerol pathway enzymes, we constructed the three different gene deletion strains *stl1*∆*, gut1*∆, and *gut2*∆ in the CBS 6412-13A haploid genetic background. All three deletions resulted in complete abolishment of glycerol growth (Figure 3), showing that the major glycerol catabolic pathway in this strain is the same as in the studied laboratory strains.

### The glycerol^+^ phenotype of CBS 6412-13A is a quantitative trait

The knowledge of the genetic determinants underlying the glycerol^+^ phenotype of CBS 6412-13A is of great interest as it might provide a profound basis for future engineering of *S. cerevisiae* for more efficient glycerol utilization. In a first step towards the identification of the genetic basis, we investigated whether the difference in glycerol growth is caused by a single polymorphism (Mendelian trait) or by multiple polymorphisms (quantitative trait). For this purpose, a hybrid strain obtained by mating CEN.PK113-1A (glycerol^-^) and CBS 6412-13A (glycerol^+^) was sporulated, and 702 single segregants were isolated using a micromanipulator and characterized for glycerol growth. The segregants were first subjected to a semi-quantitative pre-screening on solid synthetic glycerol medium. The overall data from this pre-screening showed a continuous distribution of glycerol growth phenotypes (data not shown). Among the 702 segregants, 286 did not show any growth, while 50 showed growth similar to CBS 6412-13A. The latter segregants were then subjected to a comprehensive quantitative screening in liquid synthetic medium using the Growth Profiler. Only seven of the screened segregants exhibited the same or even slightly better growth (in terms of μ_max_ and lag phase) as compared to CBS 6412-13A.

### Genetic linkage analysis between the glycerol^+^ phenotype and the *STL1*, *GUT1,* and *GUT2* alleles from CEN.PK113-1A and CBS 6412-13A

The major pathway for glycerol utilization in *S. cerevisiae* constitutes of Stl1, Gut1, and Gut2 (as also confirmed for CBS 6412-13A in this study). We sequenced the corresponding alleles in CEN.PK113-13D and CBS 6412-13A in order to check whether polymorphisms are present which could cause the differential glycerol growth phenotype. A number of polymorphisms were identified in both the coding regions and the regulatory sequences (promoter and terminator regions) of all three genes. The polymorphisms within the coding regions lead to one amino acid exchange (CBS 6412-13A versus CEN.PK113-1A) in the Stl1 protein (F193L), and four amino acid exchanges in the Gut1 protein (K28E, G107S, I193T, and E670Q). No amino acid exchange was found in Gut2.

In the following, we performed linkage analysis to determine whether these polymorphisms are among the ones contributing to the observed differences in glycerol growth between CEN.PK113-1A (glycerol^-^) and CBS 6412-13A (glycerol^+^). We selected 17 segregants in addition to the 7 which exhibited the same or slightly better glycerol growth as compared to CBS 6412-13A. Glycerol growth of these additionally selected segregants was comparable to CBS 6412-13A (μ_max_ ≥0.08 h^-1^ and lag phase ≤35 hours). The increased number of selected segregants together with the stringency of the cut-offs should have sufficient statistical power to identify the loci involved in the phenotype [33,35]. Diagnostic allele-specific PCR primers were designed, and the 24 glycerol^+^ segregants were checked whether they contained either the CEN.PK113-1A or CBS 6412-13A allele of *STL1*, *GUT1*, and *GUT2* (Table 1). The probability of random distribution was high for *STL1* and *GUT2* (*P* value >0.05), while this value was low for *GUT1* (*P* value <0.05). These data imply that there is significant linkage between the glycerol^+^ phenotype and the genetic region containing *GUT1* from CBS 6412-13A.

**Table 1:** **Results of the genetic linkage analysis for *STL1*, *GUT1*, and *GUT2***

**Gene**	**Association percentage**	***P *** **value**
*STL1*	63%	3.1 × 10^-1^
*GUT1*	83%	1.5 × 10^-3^
*GUT2*	46%	8.4 × 10^-1^

For each gene the association percentage and *P* value are shown. The association percentage represents the percentage of segregants (out of 24 superior glycerol^+^ segregants obtained from the cross between CEN.PK113-1A and CBS 6412-13A) containing the respective allele from CBS 6412-13A. The *P* value was calculated using an exact binomial test with a confidence level of 95%.

### Reciprocal hemizygosity analysis for *GUT1*

**Figure 4:**
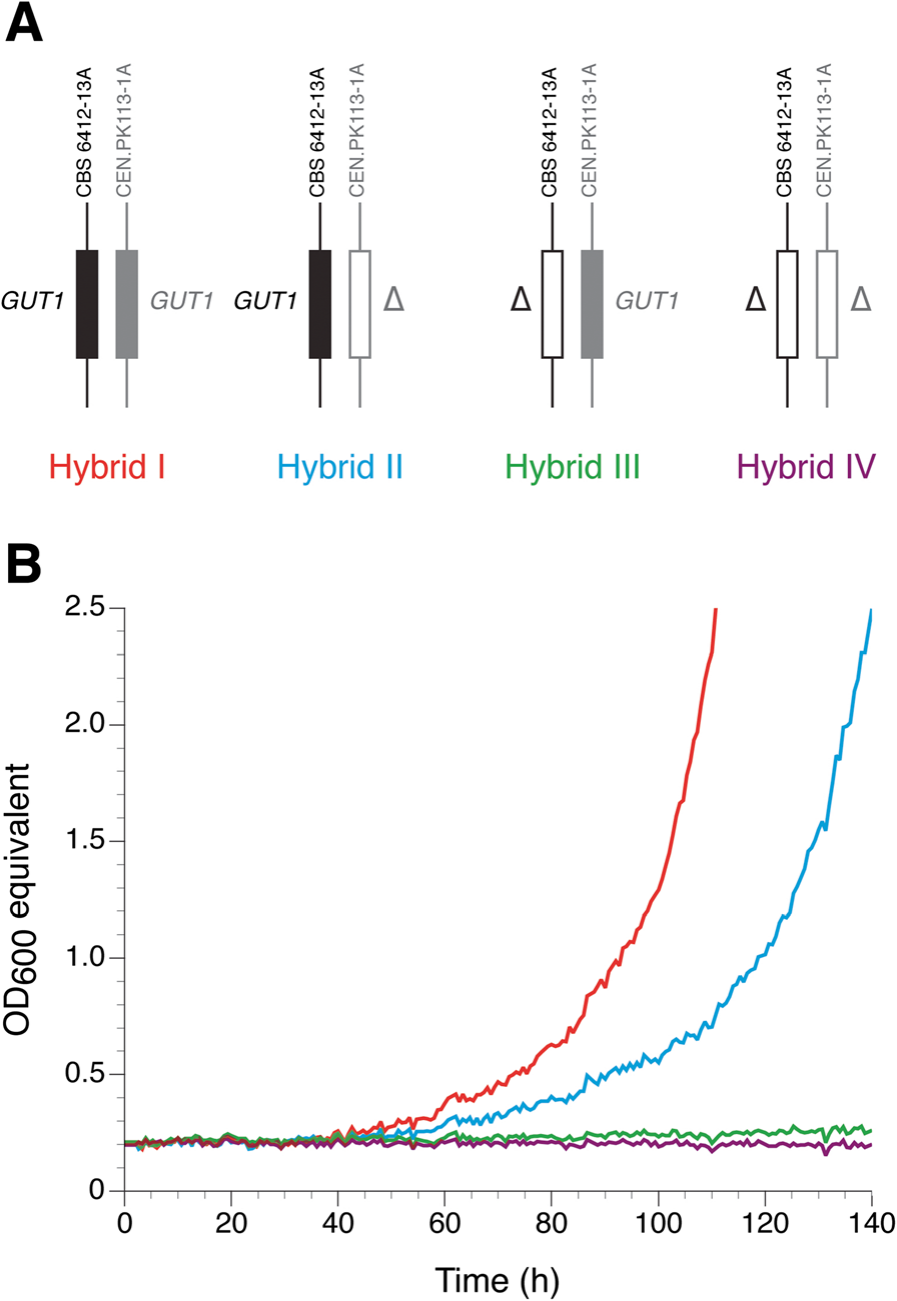
**Reciprocal hemizygosity analysis for *GUT1*. (A)** Genetic background of the four diploid hybrids constructed by mating wild-type and *gut1* deleted CBS 6412-13A and CEN.PK113-1A, respectively. All strains only differ genetically by the alleles of *GUT1*, that is, they carry either both alleles (*GUT1*_*CBS 6412-13A*_ and *GUT1*_*CEN.PK113-1A*_), one allele (*GUT1*_*CBS 6412-13A*_ or *GUT1*_*CEN.PK113-1A*_), or no alleles. **(B)** Growth of the hybrid strains in synthetic glycerol medium: hybrid I (red), hybrid II (blue), hybrid III (green), and hybrid IV (purple). Data from one representative experiment out of three independent biological replicates are shown.

The results from the linkage analysis indicated that the genetic region containing *GUT1* is linked to the glycerol^+^ phenotype. However, they do not clarify whether the polymorphism(s) found within the *GUT1* allele itself contribute(s) to the different glycerol growth phenotype, or if the causative polymorphism(s) reside(s) in a co-inherited region located in close proximity to *GUT1*. A generally accepted strategy to study whether a certain candidate gene is involved in a polygenic trait is reciprocal hemizygosity analysis [36]. In this assay, the impact of the two parental alleles of the respective candidate gene is analyzed in the same genetic background, which is the diploid hybrid strain. The allele under investigation (*GUT1*_*CEN.PK113-1A*_ or *GUT1*_*CBS 6412-13A*_) is present in one copy while the other allele is deleted (Figure 4A). In contrast to simple allele replacement in the genetic background of a haploid parent strain, reciprocal hemizygosity analysis allows study of the two different alleles in a complex genetic environment where any important interactions between *GUT1* and the various additional parent strain-specific genetic factors contributing to the superior (glycerol^+^) phenotype are retained. Our results show that the hybrid strain carrying only *GUT1*_*CBS 6412-13A*_ grew in synthetic glycerol medium, while the strain carrying only *GUT1*_*CEN.PK113-1A*_ did not grow during a time period of 140 hours (Figure 4B). This result confirms that *GUT1*_*CBS 6412-13A*_ is indeed one of the causative determinants for the glycerol^+^ phenotype of CBS 6412-13A.

Although *GUT1*_*CEN.PK113-13D*_ alone did not result in any growth, we observed that the hybrid carrying both alleles grew better than the one carrying only *GUT1*_*CBS 6412-13A*_ (Figure 4B). However, all hybrid strains showed reduced growth as compared to the haploid CBS 6412-13A strain as visible when comparing Figures 3 and 4B.

## Discussion

The development of glycerol-based bioprocesses for the production of bulk and high value fine chemicals using *S. cerevisiae* as a platform is of high commercial interest but has been hampered by the lack of comprehensive knowledge about the bottlenecks of glycerol utilization in this species. In particular, the addition of growth-supporting supplements to culture media seems to have obscured the main limitations for glycerol metabolism in this organism and therefore might have impeded their identification. In this study we confirm that the prototrophic versions of the commonly used *S. cerevisiae* laboratory strains CEN.PK113, W303, and S288c cannot grow in synthetic glycerol medium without the addition of any supplements. In contrast, a screening of a range of natural and industrial *S. cerevisiae* isolates revealed a high diversity concerning this phenotype, with some of the isolates showing growth rates of up to 0.15 h^-1^. These values are the highest ever reported for a wild-type *S. cerevisiae* strain growing in synthetic glycerol medium without any supplements, and so far have only been obtained for strains by means of evolutionary engineering. In a study of Merico *et al*. [20], glycerol growth had only been observed after prolonged cultivation periods of more than 300 hours, and subsequent enrichment by sequential cultivations under the same conditions yielded a mutant strain with a growth rate of 0.17 h^-1^. Even slightly higher growth rates of up to 0.22 h^-1^ have been obtained for several tested *S. cerevisiae* strains already after ten sub-cultivations (equivalent to about 50 generations) in an evolutionary engineering experiment reported by Ochoa-Estopier *et al*. [21]. Both the identified natural isolates as well as the evolutionary engineered laboratory strains reflect the evolutionary potential of the species *S. cerevisiae* for glycerol growth. As the *S. cerevisiae* isolates screened within the current study almost solely originate from human-associated environments (for example apple vinegar and wineries), strains with even better glycerol growth than reported here might exist in other ecological niches.

A puzzling aspect of the growth in synthetic glycerol medium is the extraordinarily long lag phase observed for all analyzed *S. cerevisiae* isolates, even for those strains showing a relatively high growth rate after entering exponential growth phase. Transcriptional changes induced by the shift in carbon source are not expected to result in such a long adaptation phase. Roberts and Hudson [37] showed that the genes encoding for the main glycerol utilization pathway (*STL1*, *GUT1*, and *GUT2)* as well as other differentially expressed genes are strongly upregulated within 30 minutes after changing the carbon source from glucose to glycerol. Although this work had been carried out in complex medium it is unlikely that the absence of supplements in the medium used for the current study would cause such a remarkable delay in regulating global gene expression. Recent work published by our group found that the prolonged lag phase is accompanied by defects in mitochondrial distribution and inheritance associated with increased mitochondrial oxidation [38]. Interestingly, toward the end of the lag phase directly preceding entry into exponential growth phase, mitochondria become more reducing and mitochondrial inheritance into the bud is restored. Though we are currently unable to assess whether mitochondrial function and dynamics are key mediators or downstream targets of the prolonged lag phase, this work presents insight on the physiological adaptation occurring during the transition to growth on glycerol.

The supportive effect of medium supplements to glycerol growth in synthetic medium is not completely clear. Their presence seems to be important for glycerol growth of many strains, and Merico *et al*. [20] suggested an improved cytosolic NAD^+^/NADH balance as the main reason for the enhanced growth. They argue that in such media the biosynthesis of amino acids becomes obsolete so that no excess cytosolic NADH is accumulated. Their hypothesis was based on the finding that also the addition of the redox sink acetoin resulted in improved glycerol growth even in the absence of amino acids.

Oddly enough, all previous published studies regarding the genetics and molecular biology of glycerol utilization in *S. cerevisiae* have been carried out using poorly growing laboratory strains and/or in the presence of medium supplements. In the beginning of our study, we could not exclude the presence of alternative functional glycerol transporters or catabolic enzymes in the glycerol^+^ strain CBS 6412 isolated in the current work, which could have been obtained by means of horizontal gene transfer from yeast species growing much better on glycerol, or a loss of certain genes in laboratory strains. Gene losses have contributed more often to yeast genome evolution [39]. However, clear indications for horizontal gene transfer events in the evolution of *S. cerevisiae* have also been provided [40-42]. In this regard, it was interesting to find that glycerol utilization in CBS 6412 relies on the same pathway (Stl1, Gut1, and Gut2) as reported for the previously characterized laboratory strains.

The identified difference in glycerol growth between the strains CBS 6412-13A and CEN.PK113-1A is a valuable starting point to identify the underlying molecular basis for this phenotype. However, a comparative gene expression analysis (including ‘omics’ technologies) or enzyme activity measurement is not feasible, since the reference strain CEN.PK113-1A does not grow in synthetic medium without supplements. Moreover, it can be expected that a whole-genome re-sequencing of both strains would reveal a high number of polymorphisms [33], and the sole knowledge of all these polymorphisms is certainly not sufficient to extract the ones determining the glycerol growth phenotype. The most efficient method to distinguish between phenotype-relevant and phenotype-irrelevant polymorphisms is genetic linkage analysis [43].

The phenotypic analysis of segregants from the cross between CBS 6412-13A and CEN.PK113-1A revealed that the glycerol^+^ phenotype is a quantitative trait, that is, several unlinked causative genetic polymorphisms are involved. Before initiating a genome-wide genetic linkage analysis, we first focused on the three glycerol utilization pathway genes. We found significant linkage of the glycerol^+^ phenotype with *GUT1*_CBS 6412-13A_, and the contribution of this gene to the phenotype was confirmed by reciprocal hemizygosity analysis. However, besides *GUT1* several other genetic loci must contribute to the superior phenotype. At first, sporulation of the CEN.PK113-1A/CBS 6412-13A hybrid did not result in a 2/2 segregation of the glycerol^+^ phenotype as would be expected if *GUT1* was the only contributing factor. Another indication is that all hybrid strains constructed for the reciprocal hemizygosity analysis showed remarkably reduced growth as compared to the haploid glycerol^+^ segregant CBS 6412-13A. These results suggest that this segregant carries additional recessive, causative polymorphisms which only contribute to the glycerol^+^ phenotype in the CBS 6412 genetic background. Moreover, we identified a few segregants from the CEN.PK113-1A/CBS 6412-13A hybrid which showed the glycerol^+^ phenotype, but carried the *GUT1* allele from CEN.PK113-1A.

There are several indications that *GUT1*_*CEN.PK113-1A*_ is functional, although the hybrid strain carrying only this allele did not grow on glycerol. The presence of *GUT1*_*CEN.PK113-1A*_ in addition to *GUT1*_*CBS 6412-13A*_ in the hybrid background enhanced glycerol growth in comparison to the hybrid harboring only the latter allele. Moreover, the haploid CEN.PK113-7D grew in synthetic glycerol medium supplemented with CSM, showing that the pathway for glycerol utilization is active in this strain. Another interesting conclusion that can be drawn from the above-mentioned result from the reciprocal hemizygosity analysis is that the total cellular Gut1 activity seems to influence glycerol growth.

Liu *et al*. [44] have shown that expression of *STL1* under the control of the strong *TEF1* promoter leads to improved glycerol growth. In our study we did not find linkage between glycerol growth and *STL1*. However, this does not exclude that Stl1 activity is one of the many factors contributing to the difference in glycerol growth between CBS 6412-13A and CEN.PK113-1A. A higher Stl1 activity is not necessarily associated with polymorphisms within the Stl1 encoding gene, but may be caused by molecular factors involved in regulating transcription, mRNA stability, translation, or post-translational modifications.

## Conclusion

In the view of using glycerol as a carbon source in industrial applications, it is a promising result that certain *S. cerevisiae* strains are able to grow in synthetic glycerol medium without complex supplements. Although crude glycerol is a rather undefined medium and usually contains a number of impurities (for example methanol, salts, soaps, heavy metals, and residual fatty acids), the presence of amino acids or nucleic bases has not been reported in this context [6]. It is clear that the glycerol growth parameters described in this study do not yet match the performance requirements for a commercial production strain. However, the knowledge about the bottlenecks of glycerol growth in *S. cerevisiae* will be a valuable starting point for further rational improvements of these isolates or of already available commercial production strains. Therefore, our ongoing studies focus on the genome-wide identification of the multiple polymorphisms (in addition to the one(s) in *GUT1*) contributing to the glycerol^+^ phenotype of CBS 6412-13A by applying quantitative trait loci (QTL) mapping [43].

## Methods

### Strains and cultivation conditions

All *S. cerevisiae* strains used in this study are listed in Table 2 and Additional file 1. The medium used for yeast strain maintenance was YPD containing 1% (w/v) yeast extract, 2% (w/v) peptone, and 2% (w/v) glucose. Yeast cells were routinely cultured at 30°C and orbital shaking at 200 rpm. All experiments were performed in synthetic medium according to Verduyn *et al*. [45] containing per liter: 5 g (NH_4_)_2_SO_4_, 3 g KH_2_PO_4_, 0.5 g MgSO_4_.7H_2_O, 15 mg EDTA, 4.5 mg ZnSO_4_.7H_2_O, 0.84 mg MnCl_2_.2H_2_O, 0.3 mg CoCl_2_.6H_2_O, 0.3 mg CuSO_4_.5H_2_O, 0.4 mg NaMoO_4_.2H_2_O, 4.5 mg CaCl_2_.2H_2_O, 3 mg FeSO_4_.7H_2_O, 1 mg H_3_BO_3_, and 0.1 mg KI. Filter sterilized vitamins were added after heat sterilization of this medium. Final vitamin concentrations per liter were: 0.05 mg D-(+)-biotin, 1 mg D-pantothenic acid hemicalcium salt, 1 mg nicotinic acid, 25 mg myo-inositol, 1 mg thiamine chloride hydrochloride, 1 mg pyridoxine hydrochloride, and 0.2 mg 4-aminobenzoic acid. The carbon source added to the medium was either 2% (w/v) glucose or 6% (v/v) glycerol. The pH was adjusted to 6.5 with 2 M KOH for the synthetic glucose medium, and to 4.0 with 2 M H_3_PO_4_ for the synthetic glycerol medium. Glycerol growth was also checked at pH 6.5, however the lag phase was found to be significantly shorter at pH 4.0 (data not shown). In order to test the impact of amino acids and nucleic bases on glycerol growth, a ready-to-use mixture of such supplements was used. In detail, 0.77 g/L CSM-URA (QBiogene, West Montreal, QC, Canada) and 0.15 g/L uracil were added to the medium. For the preparation of solid media, 3% (w/v) agar was added. In case of solid synthetic glycerol medium the agar was washed. This was necessary to significantly reduce background growth of *S. cerevisiae*, which was observed on solid medium without addition of any carbon source and thus caused by unknown components present in the agar. The washing was performed by mixing the agar powder in deionized water and decanting the supernatant after the agar had settled. This procedure was repeated three times.

**Table 2:** ***S*. *cerevisiae *** **strains used in this study**

**Strain**	**Genotype, description**	**Reference**
S288c	*MAT*a (prototrophic)	Mortimer and Johnston [46]
W303-1A	*MAT*a (prototrophic)	Thomas and Rothstein [47]
CEN.PK113-7D	*MAT*a (prototrophic)	van Dijken *et al*. [48]
CEN.PK113-1A	*MAT*α (prototrophic)	P Kötter (Euroscarf)
CBS 6412	Wild-type, diploid	CBS strain collection
CBS 6412-13A	*MAT*a, haploid segregant of CBS 6412	This study
CBS 6412-13A *gut1*∆	*MAT*a *gut1::loxP-kanMX-loxP*	This study
CBS 6412-13A *gut2*∆	*MAT*a *gut2::loxP-ble-loxP*	This study
CBS 6412-13A *stl1*∆	*MAT*a *stl1::loxP-ble-loxP*	This study
CEN.PK113-1A *gut1*∆	*MAT*α *gut1::loxP-kanMX-loxP*	This study
Hybrid I	*MAT*a/α; cross of CBS 6412-13A and CEN.PK113-1A	This study
Hybrid II	*MAT*a/α; cross of CBS 6412-13A and CEN.PK113-1A *gut1*∆	This study
Hybrid III	*MAT*a/α; cross of CBS 6412-13A *gut1*∆ and CEN.PK113-1A	This study
Hybrid IV	*MAT*a/α; cross of CBS 6412-13A *gut1*∆ and CEN.PK113-1A *gut1*∆	This study

The 52 *S. cerevisiae* strains used in the initial screening for glycerol growth are summarized in Additional file 1. Euroscarf, European *Saccharomyces cerevisiae* archive for functional analysis.

*Escherichia coli* DH5α cells carrying the vectors pUG6 or pUG66 were grown in Luria-Bertani (LB) medium (1% peptone, 0.5% yeast extract, 1% sodium chloride, pH 7.0) with 100 mg/L ampicillin. Liquid cultures of *E. coli* cells were routinely carried out in an orbital shaker at 250 rpm and 37°C. The plasmids pUG6 and pUG66 were isolated from *E. coli* cells by using a commercial miniprep kit (Qiagen, Hilden, Germany).

### Quantitative analysis of *S. cerevisiae* glycerol growth in the Growth Profiler 1152

For pre-culture, 4 mL of synthetic glucose medium in a glass tube were inoculated using cells from a YPD plate and incubated overnight at 200 rpm in an orbital shaker. The pre-culture was used to inoculate 4 mL of fresh synthetic glucose medium to an optical density (OD_600_) of 0.2 (equivalent to 4 × 10^5^ cells per mL). This culture (in the following referred to as intermediate culture) was subsequently grown under the same conditions for 48 hours. An appropriate amount of cells of the intermediate culture (to obtain an OD_600_ of 0.2 in 5 mL) was pelleted by centrifugation at 845 *g* for 5 minutes, and washed once by resuspending in 750 μL synthetic glycerol medium. After an additional centrifugation step, the cells were resuspended in 5 mL synthetic glycerol medium. An aliquot (750 μL) of this culture was transferred immediately into a well of a white Krystal 24-well clear bottom microplate (Porvair Sciences, Leatherhead, UK). Growth was recorded using the Growth Profiler 1152 (Enzyscreen, Haarlem, The Netherlands) at 30°C and orbital shaking at 200 rpm. The Growth Profiler was set to generate a scan of the plate every 40 minutes. Based on this scan, the Growth Profiler software allows to calculate the density of the cultures in each single well of a plate (green value; G-value). A calibration curve was generated in order to convert the G-values into OD_600_ values (referred to here as OD_600_ equivalents). The following equation was obtained from the calibration curve, and used throughout the study: OD_600_ equivalent = 6.1108E-09 × G-value^3.9848^.

### Mating, sporulation, and segregant isolation

Mating and sporulation were carried out according to standard procedures [49]. Tetrads were dissected using a micromanipulator from Singer Instrument Co Ltd (Watchet, UK). Mating types were determined by diagnostic PCR for the *MAT* locus [50].

### Construction of *stl1*∆, *gut1*∆, and *gut2*∆ deletion mutants of CBS 6412-13A

The *GUT1* gene was deleted in CBS 6412-13A using a cassette conferring resistance to G418 as a selectable trait, while a cassette conferring phleomycin resistance was used to delete *GUT2* and *STL1*. The deletion cassettes were PCR-amplified from pUG6 and pUG66 [51,52], respectively, with the corresponding *gene-loxP-fw* and *gene-loxP-rv* primers (Table 3) using a Phusion High-Fidelity DNA Polymerase (Thermo Fisher Scientific, Waltham, MA, USA). The primers contain a sequence complementary to the loxP sites on pUG6/pUG66 at their 3′ end and a sequence complementary to the flanking regions of the genomic integration site at their 5′ end. PCRs were performed in 50 μL and contained 10 ng of template pUG6 or pUG66, 10 mM of each dNTP, 50 pmol of each primer, and appropriate amounts of Phusion High-Fidelity DNA Polymerase and buffer according to the manufacturer’s guidelines. The following cycling parameters were used: 3 minutes of initial template denaturation at 98°C, and 30 cycles comprising a 30-second denaturation step at 98°C, a 30-second annealing step at 63°C, and a 1-minute elongation step at 72°C. The final elongation step was performed at 72°C for 3 minutes. The amplified deletion cassettes were purified from the PCR mixtures by using a PCR purification kit (Qiagen) and subsequently used for transformation of the strain CBS 6412-13A using the lithium acetate method described by Gietz *et al*. [53]. Cells were recovered in liquid YPD medium for 5 hours (30°C at 200 rpm) before plating them on YPD agar plates containing either 100 μg/mL Geneticin G418 or 20 μg/mL phleomycin.

**Table 3:** **PCR primers used to generate the cassettes for deleting *STL1*, *GUT1, *** **and *GUT2 *** **in CBS 6412-13A and for the PCR-based verification of the respective deletions**

**Gene**	**Primer**	**Sequence (5′-3′)**
*STL1*	^a^*stl1*-loxP-fw	CACTCATAGTATATAAACAAGCCCTTTATTGATTTTGAATAATTAcagctgaagcttcgtacgc
	^a^*stl1*-loxP-rv	TCAAAGCCCTCTGAAGATTTTGGGACCTGCCTCTGGAGAACAAACgcataggccactagtggatctg
	^b^verif-*stl1*∆-fw	TGGTTCACCTTTGATAGGGC
	^b^verif-*stl1*∆-rv	TGAAACTGCTTGACCTGTGG
*GUT1*	^a^*gut1*-loxP-fw	TGTGGGGGGATGCCTGTTCTCGAACCATATAAAATATACCATGTGcagctgaagcttcgtacgc
	^a^*gut1*-loxP-rv	CTAGATCTCGCAGTACTGTTTTTGGCGAATCGTGTAGCTTTTCCAgcataggccactagtggatctg
	^b^verif-*gut1*∆-fw	GTGTGGAGTAGCATAGTGAGG
	^b^verif-*gut1*∆-rv	AATGCTAGAGTCGTCAGTGCG
*GUT2*	^a^*gut2*-loxP-fw	GTCTAAAGCAAGGACTCTCCCTCCCTTATCTTGACCGTGCTATTGcagctgaagcttcgtacgc
	^a^*gut2*-loxP-rv	TTGCAAAATGGCGTCACTGTATGGGCCCGTGGCATTGACCACACAgcataggccactagtggatctg
	^b^verif*-gut2*∆-fw	TCCGATACGTTATCCACCCAA
	^b^verif-*gut2*∆-rv	TTCCTCAGCCATTTGTCTGT

^a^Gene deletion cassettes; sequences complementary to the loxP regions within the disruption cassettes are shown in lower case.

^b^Verification of gene deletions.

### Isolation of genomic DNA from *S. cerevisiae* transformants and diagnostic PCR

For the verification of disrupted genes, single cell colonies from the transformation plates were first re-streaked on plates containing the respective antibiotic and incubated at 30°C for 48 hours. Approximately 50 mg of cells from these plates were suspended in 200 μL of TE buffer (10 mMTris, 1 mM EDTA, pH 8.0). Subsequently, 300 mg of acid-washed glass beads (diameter of 0.425 to 0.6 mm) and 200 μL of phenol:chloroform:isoamyl alcohol (25:24:1) were added. The tubes were then vortexed at maximum speed for 2 minutes and centrifuged at 15,700 *g* for 10 minutes according to a protocol modified from Hoffman and Winston [54]. From the aqueous phase, 1 μL was used in a 20 μL PCR mixture containing 10 mM of each dNTP, 10 pmol of each verification primer (Table 3), and appropriate amounts of TaKaRa Ex Taq Polymerase and buffer according to the manufacturer’s guidelines (Merck KGaA, Darmstadt, Germany). The following cycling parameters were used: 3 minutes of initial template denaturation at 94°C, and 30 cycles comprising a 30-second denaturation step at 94°C, a 30-second annealing step at 58°C, and a 2-minute elongation step at 72°C. The final elongation step was performed at 72°C for 7 minutes.

### Diagnostic allele-specific PCR

Sequencing of *STL1*, *GUT1*, and *GUT2* alleles was carried out using the dideoxy chain-termination method [55] by GATC Biotech (Cologne, Germany). Diagnostic allele-specific PCR was performed according to a previously described method [35] using the allele-specific primers shown in Table 4.

**Table 4:** **Allele-specific primers used for the differentiation between the *STL1*, *GUT1, *and *GUT2 *alleles from CEN.PK113-1A and CBS 6412-13A, respectively**

**Gene**	**Primer**	**Sequence (5′-3′)**
*STL1*	Primers for specific amplification of *STL1*_*CEN.PK113-1A*_	Forward: GGTTGTTTCGCAGGTTCTCTT
		Reverse: ACTTCCTCATCATTTGGATCT
	Primers for specific amplification of *STL1*_*CBS 6412-13A*_	Forward: GGTTGTTTCGCAGGTTCTCTA
		Reverse: ACTTCCTCATCATTTGGATCC
*GUT1*	Primers for specific amplification of *GUT1*_*CEN.PK113-1A*_	Forward: GCAAACATGAGAGAAACCACA
		Reverse: AATTTCGGGCATGTGAATCAG
	Primers for specific amplification of *GUT1*_*CBS 6412-13A*_	Forward: GCAAACATGAGAGAAACTACG
		Reverse: AATTTCGGGCATGTGAATCAA
*GUT2*	Primers for specific amplification of *GUT2*_*CEN.PK113-1A*_	Forward: CGCCACTTTAGCCATTACC
		Reverse: GTGTGACAAGGTTTCAGGTTG
	Primers for specific amplification of *GUT2*_*CBS 6412-13A*_	Forward: CGCCACTTTAGCCATCACG
		Reverse: GTGTGACAAGGTTTCAGGTTA

### Reciprocal hemizygosity analysis

*GUT1* was deleted in CBS 6412-13A and CEN.PK113-1A as described above. The CBS 6412-13A and CEN.PK113-1A wild-type and *gut1* deletion strains were subsequently crossed two-by-two to obtain four diploid strains with different combinations of the *GUT1*_CBS 6412-13A_ and *GUT1*_CEN.PK113-1A_ alleles as shown in Figure 4A. The presence of the wild-type and/or deletion alleles in each diploid strain was verified by PCR as described above.

## Abbreviations

DHA: Dihydroxyacetone; CSM: Complete supplement mixture; dNTP: Deoxynucleotide triphosphate; EDTA: Ethylenediaminetetraacetic acid; EXF: Extremophilic Fungi; G-value: green value; LB: Luria-Bertani; OD: Optical density; PCR: Polymerase chain reaction; QTL: Quantitative trait loci; μmax: Maximum specific growth rate; YPD: Yeast extract peptone dextrose.

## Competing interests

The authors declare that they have no competing interests.

## Authors’ contributions

SS, MK, MC, JM, and HTTN carried out the experiments. SS, MK, and EN wrote the manuscript. All authors read and approved the final manuscript.

## Supplementary Material

Additional file 1 ***S. cerevisiae *** **strains screened for growth in synthetic medium containing glycerol as the sole carbon source.** The strains are ordered according to the time they need to reach OD_600_ of 1 as shown Figure 2A. Click here for file
