# Supplementary material for: Re-evaluation of glycerol utilization in Saccharomyces cerevisiae: characterization of an isolate that grows on glycerol without supporting supplements
Source: Biotechnol Biofuels. 2013 Nov 8;6:157. doi: 10.1186/1754-6834-6-157 (PMC3835864; doi:10.1186/1754-6834-6-157)
Supplement: Additional file 1 — S. cerevisiae strains screened for growth in synthetic medium containing glycerol as the sole carbon source. The strains are ordered according to the time they need to reach OD600 of 1 as shown Figure 2A. [file 1754-6834-6-157-S1.docx]

**Additional file 1. *S. cerevisiae* strains screened for growth in synthetic medium containing glycerol as the sole carbon source.** The strains are ordered according to the time they need to reach OD_600_ of 1 as shown Figure 2A.

| **Number** | **Strain** | **Source** | **Reference** |
| --- | --- | --- | --- |
| 1 | CBS 6412 | Sake brewing | CBS strain collection, The Netherlands |
| 2 | EXF 5040 | Straw broom for cleaning of sinks after grape pressing | Culture Collection of Extremophilic Fungi, Slovenia |
| 3 | EXF 5046 | Dry grape skins | Culture Collection of Extremophilic Fungi, Slovenia |
| 4 | EXF 4914 | Red grape skins after pressing (10 days) | Culture Collection of Extremophilic Fungi, Slovenia |
| 5 | EXF 4127 | Soil from wine cellar (wine dropping on the floor) | Culture Collection of Extremophilic Fungi, Slovenia |
| 6 | EXF 3364 | Home-made apple vinegar | Culture Collection of Extremophilic Fungi, Slovenia |
| 7 | EXF 4911 | Red grape must | Culture Collection of Extremophilic Fungi, Slovenia |
| 8 | EXF 5295 | Home-made kefir | Culture Collection of Extremophilic Fungi, Slovenia |
| 9 | EXF 3390 | Home-made apple vinegar | Culture Collection of Extremophilic Fungi, Slovenia |
| 10 | EXF 3387 | Home-made apple vinegar | Culture Collection of Extremophilic Fungi, Slovenia |
| 11 | EXF 5042 | Straw broom for cleaning of sinks after grape pressing | Culture Collection of Extremophilic Fungi, Slovenia |
| 12 | EXF 5044 | Fresh grape skins | Culture Collection of Extremophilic Fungi, Slovenia |
| 13 | EXF 4920 | First time pressed grape | Culture Collection of Extremophilic Fungi, Slovenia |
| 14 | EXF 5052 | Dry grape berries (Šmarnica) | Culture Collection of Extremophilic Fungi, Slovenia |
| 15 | EXF 3389 | Home-made apple vinegar | Culture Collection of Extremophilic Fungi, Slovenia |
| 16 | EXF 3393 | Home-made apple vinegar | Culture Collection of Extremophilic Fungi, Slovenia |
| 17 | EXF 4120 | Champagne (wine cellar Čurin) | Culture Collection of Extremophilic Fungi, Slovenia |
| 18 | EXF 4123 | Dried grape peels, remaining on wine press for 6 months | Culture Collection of Extremophilic Fungi, Slovenia |
| 19 | EXF 4126 | Wine-dry berry selection (wine cellar Čurin) | Culture Collection of Extremophilic Fungi, Slovenia |
| 20 | EXF 4122 | Pot contents below wine barrel pipe | Culture Collection of Extremophilic Fungi, Slovenia |
| 21 | EXF 5045 | Dry grape skins | Culture Collection of Extremophilic Fungi, Slovenia |
| 22 | EXF 3426 | Home-made apple vinegar | Culture Collection of Extremophilic Fungi, Slovenia |
| 23 | EXF 5247 | Harbour sea water | Culture Collection of Extremophilic Fungi, Slovenia |
| 24 | EXF 5280 | White grape skins after second pressing | Culture Collection of Extremophilic Fungi, Slovenia |
| 25 | CBS 8066 | Diploid laboratory strain^1^ | CBS strain collection, The Netherlands |
| 26 | EXF 5248 | Harbour sea water | Culture Collection of Extremophilic Fungi, Slovenia |
| 27 | EXF 5296 | Home-made apple vinegar | Culture Collection of Extremophilic Fungi, Slovenia |
| 28 | EXF 5018 | Soil in wine yard | Culture Collection of Extremophilic Fungi, Slovenia |
| 29 | EXF 5297 | Mashed pears | Culture Collection of Extremophilic Fungi, Slovenia |
| 30 | Eau de vie | Commercial strain for beverage alcohol fermentations | Wyeast Laboratories, United States of America |
| 31 | EXF 5287 | Mashed plums | Culture Collection of Extremophilic Fungi, Slovenia |
| 32 | EXF 5273 | Red wine sediment in barrel | Culture Collection of Extremophilic Fungi, Slovenia |
| 33 | Lalvin V1116 | Commercial strain for beverage alcohol fermentations | Lallemand, Canada |
| 34 | EXF 4909 | Must from below the wine press | Culture Collection of Extremophilic Fungi, Slovenia |
| 35 | EXF 5022 | Grape juice | Culture Collection of Extremophilic Fungi, Slovenia |
| 36 | EXF 5043 | Grape berries | Culture Collection of Extremophilic Fungi, Slovenia |
| 37 | EXF 4919 | Stalks white grapes | Culture Collection of Extremophilic Fungi, Slovenia |
| 38 | EXF 3391 | Home-made apple vinegar | Culture Collection of Extremophilic Fungi, Slovenia |
| 39 | EXF 3424 | Home-made apple vinegar | Culture Collection of Extremophilic Fungi, Slovenia |
| 40 | Ethanol Red | Commercial strain for bioethanol fermentations | Fermentis, France |
| 41 | Thermosacc Dry | Commercial strain for bioethanol and beverage alcohol fermentations | Lallemand, Canada |
| 42 | Alcotec 24 | Commercial strain for beverage alcohol fermentations | Hambleton Bard, United Kingdom |
| 43 | EXF 3423 | Home-made apple vinegar | Culture Collection of Extremophilic Fungi, Slovenia |
| 44 | EXF 3392 | Home-made apple vinegar | Culture Collection of Extremophilic Fungi, Slovenia |
| 45 | EXF 5294 | Red home-made wine (7 days) | Culture Collection of Extremophilic Fungi, Slovenia |
| 46 | EXF 5284 | Silage corn | Culture Collection of Extremophilic Fungi, Slovenia |
| 47 | EXF 4912 | Concrete floor in wine cellar (with wine drops) | Culture Collection of Extremophilic Fungi, Slovenia |
| 48 | EXF 4916 | Corn cob used as barrel stopper (must 24 h) | Culture Collection of Extremophilic Fungi, Slovenia |
| 49 | EXF 4925 | Mashed pears | Culture Collection of Extremophilic Fungi, Slovenia |
| 50 | EXF 5282 | Corn cob used as barrel stopper (must 24 h) | Culture Collection of Extremophilic Fungi, Slovenia |
| 51 | EXF 5054 | Soil under apple tree | Culture Collection of Extremophilic Fungi, Slovenia |
| 52 | CEN.PK | Diploid laboratory strain obtained by mating CEN.PK113-1A and -7D^2^ | This study |

^1^ The strain CBS 6088 has been the only laboratory strain reported earlier to show growth in a synthetic glycerol medium even though the growth rate of 0.010 ± 0.002 h^-1^ was very low [21].

^2^ The diploid strain CEN.PK was generated in order to be included as a reference laboratory strain that did not show any growth in synthetic glycerol medium.
